# Gap19, a Cx43 Hemichannel Inhibitor, Acts as a Gating Modifier That Decreases Main State Opening While Increasing Substate Gating

**DOI:** 10.3390/ijms21197340

**Published:** 2020-10-05

**Authors:** Alessio Lissoni, Nan Wang, Timur Nezlobinskii, Maarten De Smet, Alexander V. Panfilov, Nele Vandersickel, Luc Leybaert, Katja Witschas

**Affiliations:** 1 Department of Basic and Applied Medical Sciences—Physiology Group, Ghent University, 9000 Ghent, Belgium; alessio.lissoni@ugent.be (A.L.); antiquewn@gmail.com (N.W.); maa.desmet@gmail.com (M.D.S.); 2 Department of Physics and Astronomy, Ghent University, 9000 Ghent, Belgium; timur.nezlobinskii@ugent.be (T.N.); alexander.panfilov@ugent.be (A.V.P.); nele.vandersickel@ugent.be (N.V.); 3 Laboratory of Computational Biology and Medicine, Ural Federal University, 620075 Ekaterinburg, Russia

**Keywords:** channel gating, graphic user interface, automated analysis, transition analysis

## Abstract

Cx43 hemichannels (HCs) are electrically and chemically gated transmembrane pores with low open probability and multiple conductance states, which makes kinetic studies of channel gating in large datasets challenging. Here, we developed open access software, named HemiGUI, to analyze HC gating transitions and investigated voltage-induced HC opening based on up to ≈4000 events recorded in HeLa-Cx43-overexpressing cells. We performed a detailed characterization of Cx43 HC gating profiles and specifically focused on the role of the C-terminal tail (CT) domain by recording the impact of adding an EGFP tag to the Cx43 CT end (Cx43-EGFP) or by supplying the Cx43 HC-inhibiting peptide Gap19 that interferes with CT interaction with the cytoplasmic loop (CL). We found that Gap19 not only decreased HC opening activity to the open state (≈217 pS) but also increased the propensity of subconductance (≈80 pS) transitions that additionally became slower as compared to the control. The work demonstrates that large sample transition analysis allows detailed investigations on Cx43 HC gating and shows that Gap19 acts as a HC gating modifier by interacting with the CT that forms a crucial gating element.

## 1. Introduction

Communication between adjacent cells is in large part coordinated by gap junctions (GJs) [1]. In forming GJs, six connexin proteins oligomerize into a hexameric structure known as a connexon or connexin hemichannel (HC). Each gap junction consists of two apposed hemichannels that provide a direct pathway between the cytoplasmic compartments of adjacent cells, allowing for electrical coupling and cell–cell transfer of metabolites and signaling molecules up to ≈1.5 kDa MW [2]. Although connexin is mostly present in the form of GJs, free connexin HCs not forming part of GJs also exist and facilitate the exchange of ions and small molecules between intra- and extracellular environments [3,4]. Electrophysiological recordings show that Cx43 HCs are typically closed under resting conditions but can open in response to voltage changes, reduced extracellular Ca^2+^, elevated intracellular Ca^2+^, altered phosphorylation, and S-nitrosylation [5,6,7,8,9]. Biophysical studies further suggest that voltage-dependent Cx43 HC gating involves three distinct states: closed, fully open, and a subconductance state [5,10], with a slow gate (> 5 ms transition time) being involved in transitions between closed and fully open states, and a fast gate (< 1 ms transition time) mediating transitions to the subconductance state. These slow and fast gating events resemble those found in junctional voltage gating of GJs [11]. Interestingly, fusing a bulky EGFP tag to the C-terminal tail (CT) removes the fast gating events between the fully open and subconductance state (also called residual state) [10].

Here, we investigated the impact of CT modifications on Cx43 HC gating by making use of two approaches: fusing an EGFP tag to the CT end or supplying the Cx43 HC-blocking peptide Gap19 [6] that has been demonstrated to interact with two distinct sites on the CT [6,12]. In particular, Gap19 inhibits HC currents by perturbing CT interaction with a cytoplasmic loop (CL)-located binding site, which is necessary for Cx43 HC opening (reviewed in [13]). Making use of Gap19, we demonstrated protection against cardiac ischemia/reperfusion injury and cardiomyocyte cell death [6,14]. Gap19 is increasingly used to probe for Cx43 HC involvement in various cardiac, brain, and other disease models [7,8,15,16,17,18], making a more detailed analysis indicated and timely.

Ion channel recordings often show baseline drift and multiple levels of unitary current amplitudes, making manual analysis labor-intensive and prone to potential bias. Accordingly, most HC gating analysis has been performed on extremely limited datasets. Here, we present a new approach to analyze single-channel traces that overcomes several limitations of available programs including the inability to (i) perform transition analysis on large datasets, (ii) recognize multichannel phenotypes or subconductance, and (iii) correct fluctuating baselines automatically. We developed the software HemiGUI that includes a graphical user interface (GUI), allowing non-biased identification of gating events on the basis of transition segmentation, not requiring any a priori assumptions on channel dynamics or conductance. On the basis of this analysis, applied to a large dataset of whole-cell current recordings in HeLa cells overexpressing Cx43, we found that Gap19 inhibition of Cx43 HCs involves a 3.6-fold decrease in main state opening but was also accompanied by a 2.5-fold increase of transitions to the subconductance state that displayed significantly slowed kinetics. Slowing down CT mobility by fusing its last amino acid to EGFP slightly decreased main state transitions (9% decrease) and strongly slowed down the kinetics of main state transitions. The data support the notion that preventing CT–CL interaction with Gap19 hinders the movement of all six HC subunits to the fully open conformation.

## 2. Results

### 2.1. Analysis Flowchart and Validation

**Figure 1:**
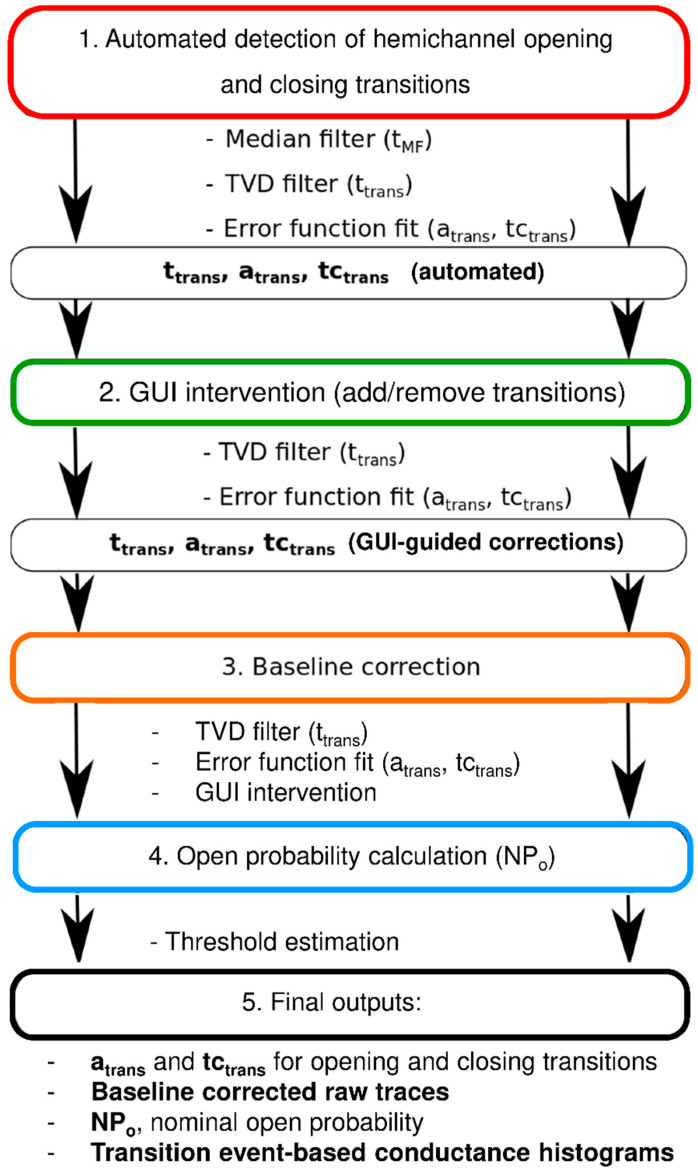
Flowchart of semi-automated analysis of unitary hemichannel currents.

Figure 1 summarizes the analysis flowchart consisting of a median filtering step, transition event detection (timing, opening, or closing), and calculation of approximate event amplitudes (for thresholding purposes). This is followed by total variation denoising and extraction of exact event time positions, amplitudes, and transition kinetics (error function fitting). From these data, an intermediate idealized trace is constructed and subtracted from the raw trace to obtain an approximation of the baseline trace. The latter is fitted to an arbitrary function that is subtracted from the raw trace to obtain drift-free raw channel activity. The transition amplitudes and kinetics are then recalculated to achieve further precision. The analysis output consists of event histograms based on transition amplitudes (a_trans_) and time constants (tc_trans_), baseline corrected raw traces, and nominal open probability NPo of whole-cell recorded channel activity (see Materials and Methods, Section 4.2).

**Figure 2:**
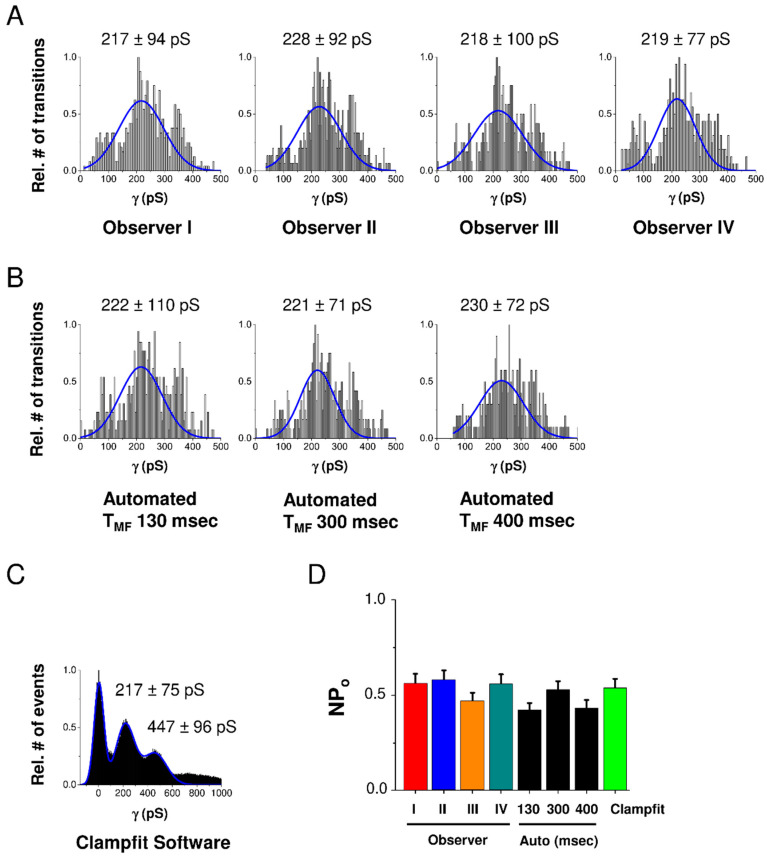
Validation of HemiGUI analysis by comparing results obtained from user intervention analysis, fully automated analysis and analysis by Clampfit software. Experiments were performed on a dataset consisting of 20 current traces randomly selected from HeLa-Cx43 whole-cell recordings involving voltage steps from −30 mV to +70 mV (*N* = 3; ***N*** = 4; *n* = 20). (**A**) All conduction transition histograms were obtained by semi-automated analysis by four independent observers. (**B**) Automated analysis and the effect of different median filter time window (T_MF_) sizes. A T_MF_ of 300 ms value based on the slowest transition time observed in the dataset gave a distribution most close to the one observed with semi-automated user intervention-based analysis. (**C**) All-point histogram generated from baseline-corrected traces generated by Clampfit. (**D**) Summary graph of nominal open probability (NPo) data demonstrating no statistically significant differences between Clampfit and the two modes of HemiGUI analysis (one-way ANOVA, Bonferroni post-test).

In order to validate the program, we compared its fully automated operation with human experience-driven intervention through the GUI and with existing Clampfit software from the pCLAMP™ 10 Software Suite provided by Axon/Molecular Devices. For validation we used a set of 20 HC current traces recorded in HeLa-Cx43 cells stepped at voltage from a resting potential of −30 mV to +70 mV. As depicted in the conductance histograms of Figure 2A–C, GUI-processed analysis performed by 4 independent users was not significantly different from the automated analysis in HemiGUI or manual analysis performed with Clampfit, as confirmed by the NPo chart in Figure 2D. We further found that increasing T_MF_ (see Material and Methods section) of the median filter improved the signal-to-noise ratio but also increased the number of missing true opening/closing events upon GUI inspection. On the basis of this, we kept the initially chosen T_MF_ of 300 ms (see the Material and Methods section), which led to a robust approximation of opening/closing events with minimal GUI intervention to eliminate false/erroneous events (mostly in the low conductance range). We next verified the robustness of automated analysis for varying the time window size used for error function fitting but found the results to be robust in the range of 130 to 400 ms (Figure 2B).

For the data analysis that follows, we consistently included GUI intervention to verify correctness of the proposed gating transitions.

### 2.2. Gating Profiles of Cx43 Hemichannels

**Figure 3:**
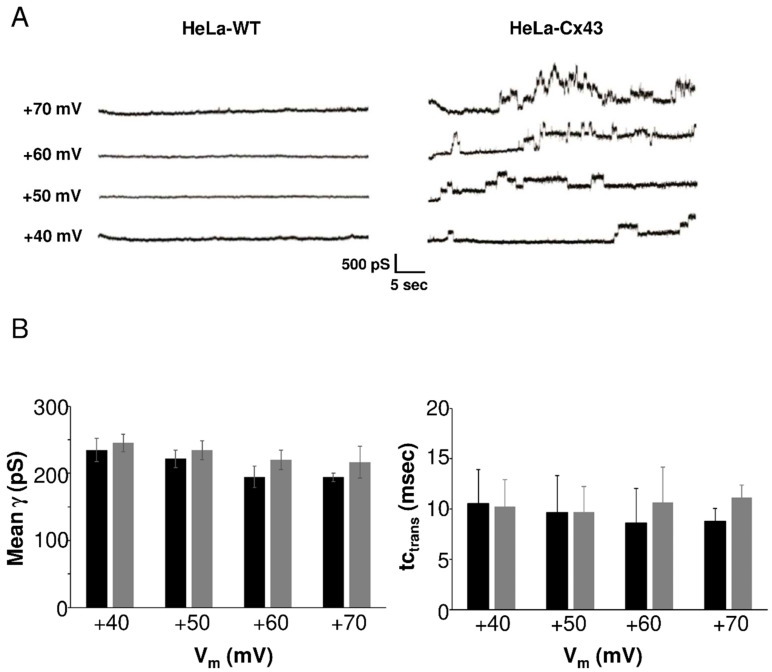
Unitary currents in HeLa-WT and HeLa-Cx43 cells. (**A**) Representative current traces in response to stepping positive membrane potentials (Vm) from −30 mV to potentials in the range of +40 to +70 mV. Unitary event activity was only observed in HeLa-Cx43. (**B**) Average unitary conductance and time constant of the transitions in HeLa-Cx43 obtained by two observers (*N* = 4; ***N*** = 15; *n* = 132, 33 traces per Vm step). There were no differences in conductance or time constants recorded at different voltages (one-way ANOVA with Bonferroni post-test).

We characterized HC currents recorded in HeLa-Cx43 cells in a larger experimental cohort. Example traces depicted in Figure 3A (right panel) showed resolvable unitary Cx43 HC events evoked by positive voltage steps from a resting potential of −30 mV to voltages in the range of +40 to +70 mV. In HeLa-WTs (left panel), current traces were flat without event activity. Unitary activity in HeLa-Cx43 displayed three distinct states: closed, subconductance (also called “residual”), and fully open states [5,10,19], as shown in Figure S4, which summarizes the states found in the overall dataset. The HC activity in HeLa-Cx43 cells was first analyzed in terms of voltage sensitivity. For that purpose, we analyzed the unitary conductance and time constant of the transitions (tc_trans_). Figure 3B summarizes average transition conductance and kinetics as a function of the stepping voltage, demonstrating that these parameters were not significantly affected by voltage. As there were no voltage-dependent differences in conductance and kinetics, we performed all further analysis on pooled traces recorded at +40, +50, +60, and +70 mV voltage steps in order to maximize the event pool.

**Figure 4:**
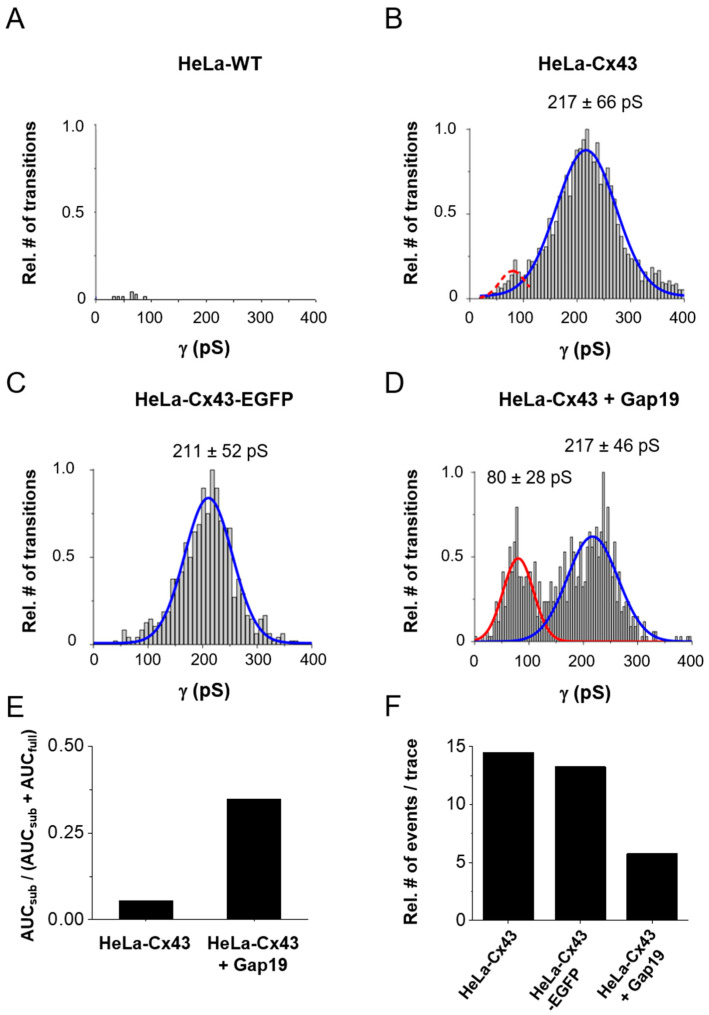
Distribution of transition conductance and the effect of CT-tagged EGFP and CT-interacting Gap19 peptide. (**A**) Histogram analysis of transitions expressed as conductance illustrating non-significant event activity in Hela-WT cells (*N* = 3; ***N*** = 9; *n* = 65, 16 traces per + 40, + 50, and + 60 mV steps and 17 traces per + 70 mV step). (**B**) HeLa-Cx43 cells showed significant event activity with a unitary conductance of the transitions centered around 217 pS (66 pS SD of the distribution) (*N* = 4; ***N*** = 15; *n* = 132, 33 traces per Vm step). (**C**) Transitions were similarly distributed in HeLa-Cx43-EGFP cells with a main peak at 211 pS (*N* = 3; ***N*** = 6; *n* = 39, 9 traces per + 40 mV step and 10 traces per + 50, + 60, and +70 mV steps). (**D**) The distribution was completely altered by exposure of HeLa-Cx43 cells to 100 µM Gap19 (*N* = 3; ***N*** = 8; *n* = 50, 12 traces per + 40 and + 50 mV steps and 13 traces per + 60 and + 70 mV steps), demonstrating a smaller main peak and the appearance of a second peak centered at 80 pS (red curve). The dashed red curve in panel (**B**) is an event frequency-adjusted scaled version of the 80 pS curve. The number of transitions were normalized to maximum observed in each cluster of the experiment. The main peaks (fully open state) were compared between the different groups by one-way ANOVA and Bonferroni post-test, resulting in no difference in conductance. (**E**) Ratios of the number of substate transitions (AUC_sub_) relative to all transitions (AUC_full_ + AUC_sub_). (**F**) All transition event counts expressed per 30 s trace for the different conditions shown.

Figure 4A,B illustrates conductance histograms in HeLa-WT and HeLa-Cx43 cells. For Cx43, the conductance followed Gaussian distribution, peaking around 217 ± 66 pS (mean ± SD) (*N* = 4; ***N*** = 15, *n* = 132) as opposed to the negative control data obtained from HeLa-WT cells shown in Figure 4A (*N* = 3; ***N*** = 9; *n* = 65). Gating to a subconductance/residual state appeared sparsely and was therefore not apparently visible in the total event distribution from Figure 4B. In HeLa-Cx43 cells with an EGFP tag attached to the CT end (Figure 4C), the conductance was comparable to non-tagged Cx43 (Figure 4B). In a next step, we analyzed the effect of the Cx43 HC-inhibiting peptide Gap19 on the unitary conductance distribution in HeLa-Cx43 cells. Gap19 interacts with two CT-located sites and thereby prevents CT interaction with the CL that is necessary for HC opening [6,12,20,21]. Interestingly, in the presence of Gap19 (100 µM, added via the pipette), an additional conductance state emerged from the recordings, displaying a pronounced 80 ± 28 pS (mean ± SD) subconductance state in addition to the main ≈217 pS open state (Figure 4D). Similar substate conductance values have been reported by us (58 pS, [5]) and others (75 pS, [10]).

To further analyze Gap19-induced changes in Cx43 HC gating, we calculated the ratios of the area under the curves (AUCs) of the 80 pS and 217 pS centered distributions in the control and in the presence of Gap19 (Figure 4E). For that purpose, we first fitted the 80 ± 28 pS Gaussian of the Gap19 condition to the event frequencies observed in the control HeLa-Cx43 dataset in that particular conductance range (red dashed curve in Figure 4B). Figure 4E shows that Gap19 increased the number of substate transitions relative to all transitions by a factor of ≈6.5. Such ratio analysis only reports relative shifts in the gating profiles and we further gauged the effect of Gap19 on the overall gating activity by counting the number of channel transitions during the 30 s recording time for each trace. Figure 4F illustrates that Gap19 decreased the transition frequency ≈2.5-fold (all transitions) compared to control recordings in HeLa-Cx43. For comparison, event activity in HeLa-Cx43-EGFP was only slightly lower compared to HeLa-Cx43 (≈91% of the event activity in HeLa-Cx43). On the basis of these data, we calculated that in the control (HeLa-Cx43), 94.7% of the transitions were to/from the main state while 5.3% were substate transitions; in the presence of Gap19, these numbers changed to 65.3% and 34.7%, respectively. In absolute terms, these values translate to 13.7 transitions per 30 s trace for the main state and 0.8 for the substate; with Gap19 these values became 3.8 and 2.0 transitions per 30 s trace, respectively. These considerations let us conclude that Gap19 reduces main state gating transitions by 3.6-fold (13.7/3.8) and increases substate gating 2.5-fold (2.0/0.8). The latter value is lower than the 6.5-fold increase found in Figure 4E, where alterations in total transition counts of Figure 4F were not taken into account.

**Figure 5:**
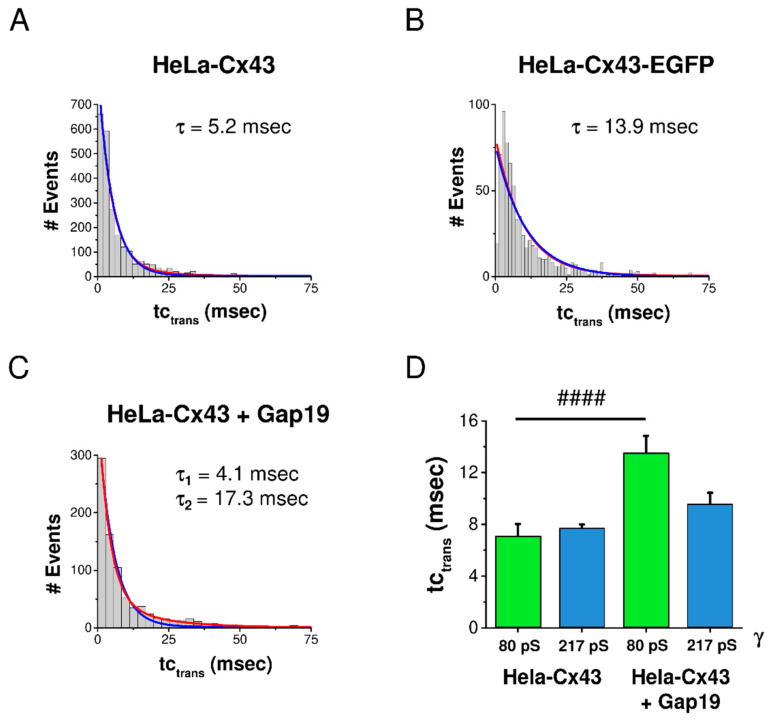
Distribution of transition kinetics and effect of CT-tagged EGFP and CT-interacting Gap19 peptide. Mono- and biexponential fits are displayed in blue and red, respectively. (**A**) In HeLa-Cx43 cells, tc_trans_ was distributed along a monoexponentially decreasing function characterized by a τ of 5.2 msec (*N* = 4; ***N*** = 15; *n* = 132). (**B**) For HeLa-Cx43-EGFP, τ was more than twice as large compared to HeLa-Cx43 (*N* = 3; ***N*** = 6; *n* = 39). (**C**) HeLa-Cx43 treated with Gap19 showed a second longer τ compared to the control (*N* = 3; ***N*** = 8; *n* = 50). (**D**) Selective analysis of tc_trans_ for transitions in the subconductance and main state demonstrated that Gap19 significantly slowed down transitions to the subconductance state while leaving main state transitions unaffected (Mann–Whitney *U* test, #### *p* ≤ 0.0001). Number of events: HeLa-Cx43 80 pS (129 events), HeLa-Cx43 217 pS (1656 events), HeLa-Cx43 + Gap19 80 pS (193 events), HeLa-Cx43 + Gap19 217 pS (453 events).

In a last step, we analyzed the effect of EGFP-tagging and Gap19 on the kinetics of Cx43 gating by analyzing tc_trans_. The degree of goodness of curve fit was checked with mono- and biexponential fits for each condition. In control HeLa-Cx43 cells, tc_trans_ was mono-exponentially distributed with a characteristic constant τ of 5.2 msec (Figure 5A). In HeLa-Cx43-EGFP, the transitions were slower with a τ value of 13.9 msec (Figure 5B). In the presence of Gap19, a second τ was evident, having a value of 17.3 msec (Figure 5C).

As Gap19 strongly enhanced gating to the subconductance state, we were interested whether this would perhaps specifically affect kinetics of subconductance transitions. For that purpose, we analyzed the effect of Gap19 on tc_trans_ for transitions in the 80 ± 28 pS and 217 ± 66 pS conductance ranges (transitions in the range of one SD above and below the mean). Interestingly, Gap19 significantly slowed down transitions to the subconductance state, causing tc_trans_ to almost double compared to control without peptide (Figure 5D). By contrast, the kinetics of transitions to the main conductance state were not altered by Gap19.

## 3. Discussion

The present work was performed with HemiGUI-based analysis software to investigate Cx43 HC gating in a large dataset of channel-opening events. Previously published data were invariably restricted to small event numbers often limited to a few channel openings. We used this approach to investigate the effect of CT tail modifications consisting of adding a bulky EGFP tag or supplying Gap19 that binds to the CT and interferes with CT–CL interaction. We demonstrated that (i) gating events to the subconductance state were rare and difficult to distinguish within a majority of gating events to the fully open state, (ii) CT tagging with EGFP slowed the opening/closing transition rates, and (iii) Gap19 decreased overall gating activity but strongly increases the propensity of subconductance transitions that also become significantly slower.

Gating of Cx channels involves both N-terminal (NT) and CT structures, with the NT being involved in voltage gating [22,23,24] and the CT in chemical gating by pH [25,26,27] and cytoplasmic Ca^2+^ [5,12,21]. Cx gating structures strongly differ from those of voltage-gated sodium or potassium channels, with presumed gating domains being involved in gating as well as permeation tasks [28]. In any case, the NT and CT structures play crucial roles, which may rely on interaction with the CL [2]. This is certainly well documented for the CT, which, when interacting with the CL, acts to close Cx43 GJs, as occurs in acidifying conditions according to a ball-and-chain model (reviewed in [29]). By contrast, the very same CT–CL interaction has an exactly opposite effect on Cx43 HCs, where it brings the channels in a state that is available for opening with electrical or chemical triggers (reviewed in [13]).

Gap19 peptide is composed of a CL sequence involved in CT–CL interaction with the CT; in fact, it is part of a larger L2 domain that is the crucial for CT–CL interaction (see Figure 1 in [13]). Consequently, supplying Gap19 peptide disrupts the CT–CL interaction by binding to the CT at two domains: the Src-homology domain 3 (SH3) and the last nine amino acids at the CT end [12,21]. Interestingly, fusing EGFP to the CT end slows down hemichannel transitions to the fully open state (Figure 5B) and so does Gap19 with the single but important difference that this only occurs for the subconductance state and not for the fully open state (Figure 5D). The slowing down of main gating transitions by EGFP has been reported by others [10,11,30] and is probably caused by a mass effect of this high-MW tag (26.9 kDa). The EGFP-loaded CT may thereby slow down conformational changes of CT-located pore-lining residues [31]. The slowing down of substate gating by Gap19 (MW 1161) is likely not the result of a mass effect but may be linked to reorganization of the CT consequent to disruption of CT–CL interaction. Gap19 not only affected transition kinetics of the subconductance state (Figure 5C) but also increased the frequency of substate gating transitions (Figure 4D,E) while decreasing overall transition frequency (Figure 4F). Thus, Gap19 disruption of CT–CL interaction decreases the availability for full opening but markedly favors substate opening. The decreased full opening activity is in line with the Cx43 HC inhibiting effects of Gap19, as previously described [7,20,32,33]. By contrast, the Gap19-induced increase in substate gating indicates that disrupted CT–CL interaction does not fully silence hemichannel activity and allows residual background activity.

In terms of substate gating, both the NT and the CT have been involved: the NT is hypothesized to move towards the cytoplasm and thereby narrow the channel pore [28,34], while the CT has been proposed to interact with the CL for substate gating, at least as studied in Cx43-based GJs [27,35]. Our present work suggests that this is different in Cx43 HCs, where disrupted CT–CL increases substate gating. The increased incidence of partial openings may be the result of an altered cooperativity of the HC subunits upon Gap19 binding to the Cx43 CT and consequent disruption of CT–CL interaction. Transition from the closed to the fully open state requires cooperated conformational change of all six subunits [36]. However, Gap19 does not necessarily bind to all six subunits to restrain the HC activation, making it conceivable that remaining subunit(s) available for CT–CL interaction may confer the increased gating to the subconductance state.

Alternatively, the present data may also be interpreted in the context of the “cork” model for Cx channel gating. The cork model has been suggested for GJs where interaction of calmodulin (CaM) with connexin at its predicted binding site in the CL results in partial or complete pore closure [37]. The CaM–cork gating model comes in two flavors: the Ca–CaM–cork mechanism includes Ca^2+^-induced CaM activation, while the CaM–Cork mechanism encompasses physical blockage of the channel vestibule without activation by Ca^2+^ [38]. Since in our experiments Ca^2+^ in the pipette solution was buffered to ≈50 nM corresponding to normal resting [Ca^2+^]_i_, the CaM–Cork model may be relevant here. We previously reported on CaM involvement in Ca^2+^-dependent activation of Cx43 HCs [39], but much less is known about CaM’s effects on HC closed states where channel availability is governed by CT–CL interaction [21]. In line with the CaM–cork gating model [40], disruption of CT–CL interaction in Cx43 HC by Gap19 may facilitate binding of CaM to the corresponding L2 domain in the CL, which overlaps with the CaM domain by nine amino acids (see Figure 1 in [13]). Assuming that the L2 region is situated in close association with the pore forming part of the channel vestibule [41], CaM would act as a gating particle, plugging the mouth of the HC upon binding. Possibly, the increased occurrence of subconductance transitions observed in our experiments was due to CaM temporarily blocking the channel pore, leading to partial opening of Cx43 HC in the presence of Gap19. Clearly, the role of CaM in the CT–CL interaction requires further investigation to build a more comprehensive and detailed gating model of Cx43 HC.

A number of studies have indicated that the subconductance state has a different permeability to larger molecules compared to the fully open state [42,43]. Thus, the substate may serve as a selectivity filter that limits the loss of metabolic substrates or signaling molecules while maintaining some flow of smaller substances. Consequently, Gap19 might exert its protective effect not only by preventing Cx43 HC main state opening but also by inducing substate transitions that allow some passage of, e.g., atomic ions or others.

In conclusion, Gap19 has a bimodal effect on Cx43 HC gating, decreasing gating to the fully open state while increasing substate gating, suggesting that Gap19 acts like a gating modifier on Cx43 HC. This is in line with our previous findings of Gap19 effects on CT–CL interaction, which are disrupted by Gap19 and thereby make the HCs less available for opening. Gap19 is thus certainly not a HC pore blocker unless it is applied at above 500 µM concentrations [5]. The work furthermore demonstrates the potential of semi-automated analysis of large gating event datasets in obtaining increased insight in Cx channel gating.

## 4. Materials and Methods

### 4.1. Electrophysiological Recordings

All experimental data were obtained with the patch-clamp technique in whole-cell recording mode on HeLa cells stably overexpressing mouse Cx43 (HeLa-Cx43) and corresponding control HeLa WT cells. The open probability of Cx43 HC in HeLa cells is low, hence the necessity to record unitary currents in whole-cell configuration [10]. Extracellular solution was composed of (in mM): 150 NaCl, 5.4 KCl, 2 CaCl_2_, 2 MgCl_2_, 2 pyruvic acid, 5 glucose, and 5 HEPES at pH 7.4, while the pipette solution consisted of (in mM): 130 CsCl, 10 sodium aspartate (NaAsp), 0.26 CaCl_2_, 1 MgCl_2_, 2 EGTA, 5 tetraethylammonium (TEA)-Cl, and 5 HEPES at pH 7.2. Whole-cell recording was performed on the same day with or without Gap19 in the pipette on the same batch of cells. Pipettes were backfilled with either pipette solution or pipette solution containing Gap19 (100 µM) using two different syringes that were kept on ice. Recordings were commenced 2 min after establishment of the whole-cell configuration. Currents were recorded with an EPC 7 PLUS patch-clamp amplifier (HEKA Elektronik, Germany). Unitary HC activities were elicited by stepping the HeLa-Cx43 cells from a holding potential of −30 mV to positive membrane potentials (Vm) ranging from +40 to +70 mV for 30 s. Data were digitized at 4 KHz using a NI USB-6221 data acquisition device (National Instruments, Austin, TX, USA) and WinWCP acquisition software designed by Dr. J. Dempster (University of Strathclyde, United Kingdom). Measured currents were filtered by a 7-pole Bessel low-pass filter at 1 KHz cut-off frequency. Unitary conductances were calculated from the elementary current transitions Δi as: γ = Δi/Vm. Cx43 HC form poorly selective pores with an E_rev_ ≈ 0 mV as described in our previous work [5,6,15].

### 4.2. Algorithm for Semi-Automated Analysis of Hemichannel Currents

The current traces were processed in different steps, allowing user intervention though a GUI. Below, we explain the different steps, which are summarized in Figure 1. Colors match the program parts depicted in Figure S1. Software code is available on GitLab (https://gitlab.com/Nezlobinsky/hemigui).

#### 4.2.1. Semi-Automated Detection of Opening and Closing Transitions

In the GUI, once the data are loaded, the raw data are shown in blue (Figure S1A,B). By clicking “find” in the GUI, the raw data are automatically filtered to find the exact times of the openings and closings of the channels (t_trans_), the amplitude of the openings (a_trans_), and the characteristic time constant of the transition event (tc_trans_). This is done as follows.

(a) Median filter

First, the raw data were filtered by a median filter with a time window of 0.3 s (T_MF_) to denoise the extended time series of recordings (30 s). This means that all the data points in each time interval of 0.3 s were replaced with the median of that data series; 0.3 s was chosen, as pilot analysis indicated 300 ms for the slowest transition time observed. As compared to low frequency filters, median filters not only remove high frequency noise from the signal but also keep baseline drift distinguishable from true opening and closing events (as compared to the low pass filter). In addition, they perform better in preserving the amplitude of each transition [44,45]. Locations of HC opening/closing events (“transitions”) are detected if the derivative of the median filtered data is larger than a certain threshold set to 60 pS (this number can be adapted in the GUI). This threshold corresponds to twice the standard deviation (SD) of background noise (30 pS) recorded in 6 random current traces of 5 s in HeLa-Cx43 clamped at −30 mV. This threshold is only applied in the initial median filter transitions detection part of the algorithm, which is used to find the main events for the purpose of background current subtraction explained in Section 4.2.3. As such, events with below 60 pS transitions will still be present and appear in the finalized analysis.

(b) TVD filter

The median filter distorts the time locations of the transitions and only delivers an approximate time position symbolized by t_MF_. A better suited filter that maintains correct time positions is total variation denoising (TVD) (see Figure S2). This filter was again applied to the raw data and effectively denoised the data without affecting the sharp transition edges in the underlying signals [46].

The regularization parameter λ plays a critical role in the denoising process. When λ = 0, there is no smoothing and the result is the same as minimizing the sum of squares. As λ approaches infinity, however, the total variation term in the TVD filter plays an increasingly strong role, which forces the result to have smaller total variation at the expense of being less like the input (noisy) signal. Thus, the choice of regularization parameter is critical to achieving just enough noise removal. The regularization parameter λ was set to 10,000 and the number of iterations to 30. In order to find the precise locations of the transitions (t_trans_), the program searched for the maximal derivative of the TVD-denoised signal within the event window delivered by the median filter, encompassing the time window [t_MF_ −300 ms, t_MF_ +300 ms].

Figure S3 shows on an expanded time scale how the transitions were fitted by the TVD filter for representative traces recorded in the three experimental conditions (HeLa-Cx43, HeLa-Cx43 + Gap19, and HeLa-Cx43-EGFP).

(c) Determining the transition amplitudes and kinetics

The amplitude of the transitions was detected by fitting the transition to the following function: f(x, A, B, C) = A erf(x/B) + C, whereby “erf” represents the error function that is given by

$$erf\left(x/B\right)=\frac{1}{\sqrt{\pi }B}\int_{-x}^{x}exp\left(-{\left(t/B\right)}^{2}\right)dt$$

and involves integration over the time window [t_trans_ −150 ms, t_trans_ +150 ms] (window size = maximum transition time, i.e., 0.3 s). In case there was another jump in the fitting window, the region is automatically readjusted by the program until the extra jump does not affect the fitting.

The amplitude of the transition (a_trans_) is then defined as 2*A, while the characteristic time constant of the transition event (tc_trans_) is given by the parameter B. The amplitude of each transition a_trans_ is depicted on the raw trace in the GUI (red for openings, green for closings), as illustrated in Figure S1A,B.

Parameter C is a constant equal to the mean baseline values (*y*-axis) at the time interval considered for the baseline correction.

#### 4.2.2. User Intervention through the GUI

For each trace, the software automatically detects opening and closing events. However, events may be missed or wrongly detected. The GUI allows for the manual removal of erroneous events (“Del positive”, “Del negative”) or addition of missed events (“Add positive”, “Add negative”). For the addition of new events, the program HemiGUI automatically searches for the steepest derivative (as in Section 4.2.1 (b)) within a manually defined time window (standard set as 500 data points) on the TVD-filtered data. The position, amplitude, and kinetics of newly defined events are then calculated and added as described in Section 4.2.1 (b) and (c).

Figure S1A illustrates the GUI screen and an example trace, displayed as a raw trace (blue) and a recalculated trace after removal of all events on the basis of subtraction of transition amplitudes over the duration of the event (orange trace). In addition, “Display tvd” can also help to visualize the jumps. Zooming in and freely scrolling within a trace for better observation of the transitions is possible by pressing “Interact”. By doing so, it is possible to inspect the trace in greater detail (purple dashed square in Figure S1A) and correct missed or false-positive transitions.

#### 4.2.3. Baseline Correction

Baseline correction is performed as follows: first, channel events are removed by subtraction of the transition amplitudes over the duration of the event (orange trace in Figure S1). Next, the trace is fitted to one of the following functions according to a non-linear least square method:

1. a single exponential function: f(x, a, b, c) = a(1 − exp(-x/b)) + c
2. a double exponential function: f(x, a, b, c, d, e) = a(1 − exp(-x/b)) + c(1 − exp(-x/d)) + e
3. a logarithmic function: f(x, a, b, c) = a ln(x + c) + b
4. a linear function: f(x, a, b) = a x + b

The baseline-corrected trace is then reanalyzed involving TVD filtering to obtain t_trans_, error function fitting to obtain a_trans_ and tc_trans_, and GUI intervention allowing further correction of missed or erroneous events. This process is repeated each time the baseline fit is updated.

#### 4.2.4. Determination of Open Probability

Channel activity is expressed as nominal open probability NPo (number of active channels in whole-cell recording x open probability), since it is difficult to determine the number of channels present in the membrane. For each trace, NPo was calculated from the ratio of the open time and recording time (30 s). NPo values were determined after setting the open-closed discriminator (threshold) half-way between the baseline and fully open state levels.

In the graphical interface the open state threshold is indicated by a red line drawn on the yellow trace in Figure S1A. Transitions above this threshold are used to calculate the total open time of the channels over the duration of the trace. This threshold can be manually adapted by the user in the GUI. Time spent by the channels in any open state is indicated by the black lines in the lower panel of Figure S1A below the yellow trace. 

In the Clampfit calculations (Figure 2C,D), NPo was determined from the ratios of the area under the peaks fitted by a Gaussian distribution within a mean ± SD area, after manual correction of the baseline. Clampfit version 10.7 was used (Molecular Devices, San Jose, USA).

### 4.3. Statistical Analysis

Data are expressed as mean ± SEM, with *n* giving the number of traces, ***N*** the number of cells, and *N* the number of experiments.

Two groups were compared with a non-parametric Mann–Whitney *U* test. Multiple groups were compared by one-way analysis of variance and a Bonferroni post-test, making use of OriginLab software (OriginLab Corporation, Northampton, USA). Results were considered statistically significant when *p* ≤ 0.05 (# for *p* ≤ 0.05, ## for *p* ≤ 0.01, ### for *p* ≤ 0.001, and #### for *p* ≤ 0.0001).

## Supplementary Materials

Supplementary materials can be found at https://www.mdpi.com/1422-0067/21/19/7340/s1. Figure S1. The graphic user interface (GUI). Figure S2. Single channel detection strategy. Figure S3. TVD fitting of representative transitions obtained in three experimental conditions. Figure S4. Schematic representation of gating states observed in this study.
