# Supplementary material for: Gap19, a Cx43 Hemichannel Inhibitor, Acts as a Gating Modifier That Decreases Main State Opening While Increasing Substate Gating
Source: Int J Mol Sci. 2020 Oct 5;21(19):7340. doi: 10.3390/ijms21197340 (PMC7583728; doi:10.3390/ijms21197340)
Supplement: Supplementary file 1 [file ijms-21-07340-s001.pdf]

Supplementary Figures:

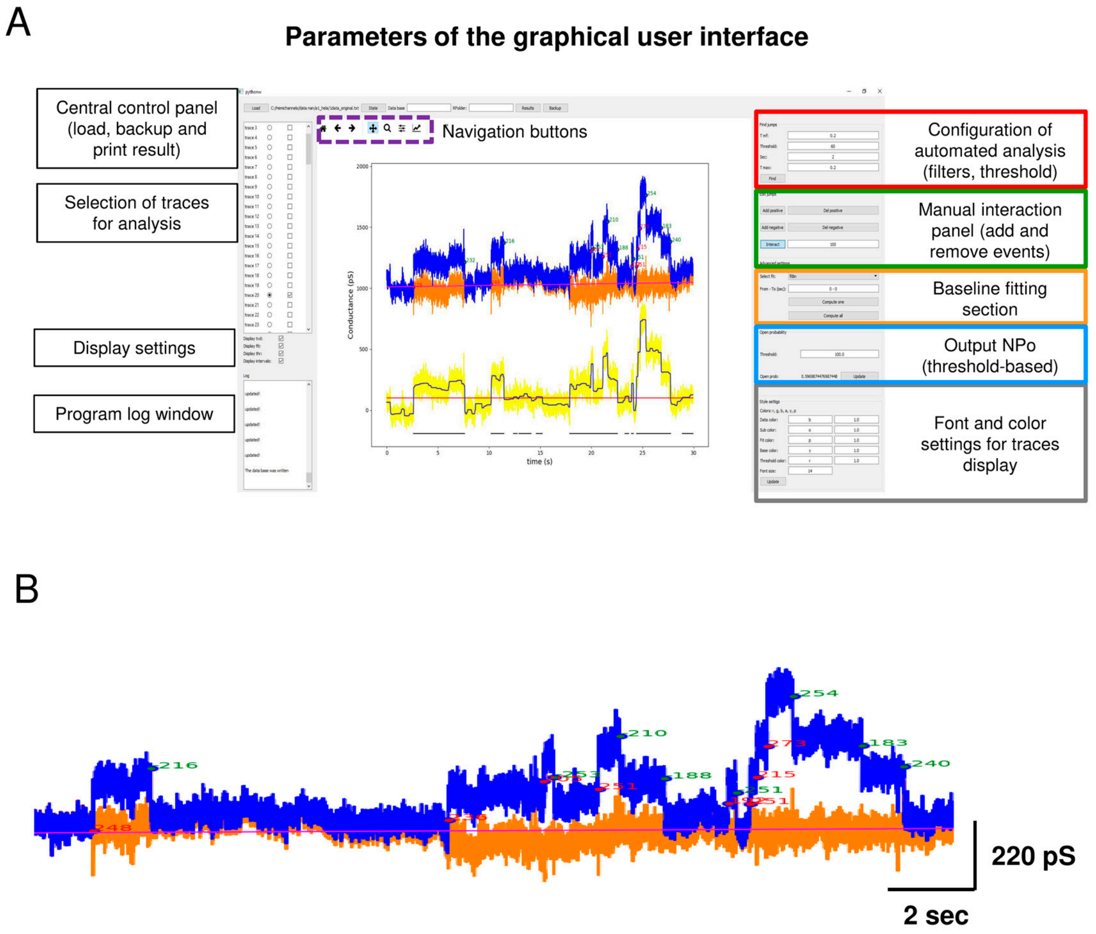

Supplementary Figure S1. The graphic user interface (GUI). A. Screenshot of the interface showing a raw trace (blue) and a baseline (orange). The yellow trace below is the baseline-corrected trace after the fitting and the red line is the threshold level for NP_o calculation (lower panel). Above this threshold channels are considered open, indicated by black lines below the yellow trace. Summation of open times in relation to the total time of the trace gives the NP_o. **B.** Enlargement of blue and orange trace of panel **A.** The blue trace is the raw trace while the orange trace is a recalculated version after removal of all events based on subtraction of transition amplitudes over the duration of the event. Transition event amplitudes expressed as conductance values (pS) are added to each event as red (opening) and green (closing) numbers on the blue trace.

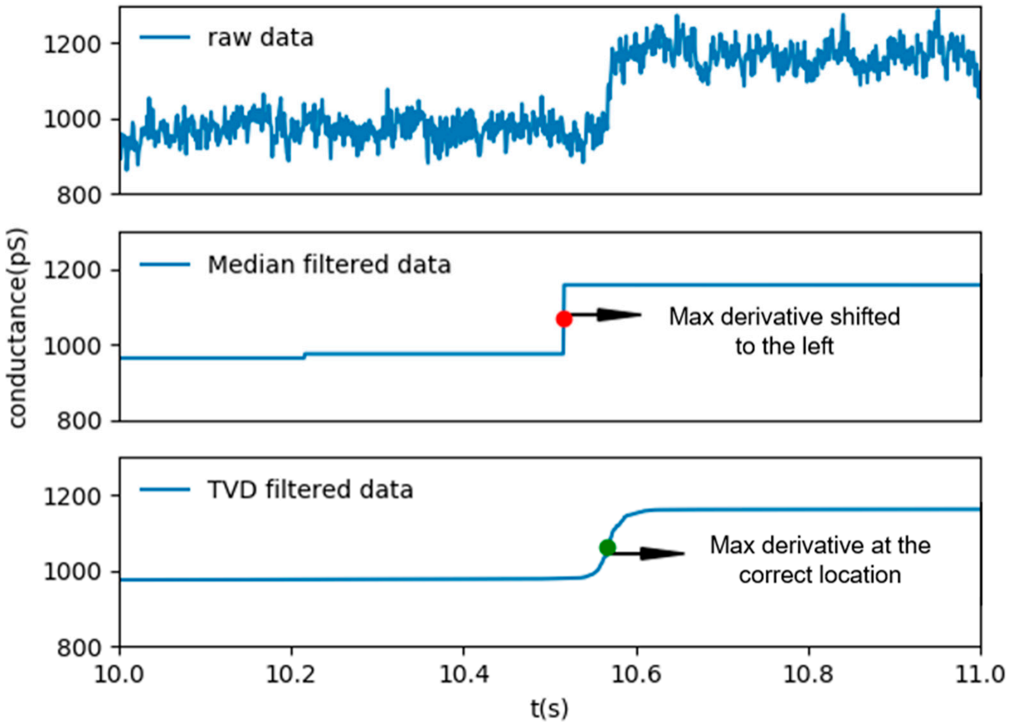

Supplementary Figure S2. Single channel detection strategy. Strategy for detection of the jumps in the raw data (upper trace). Middle trace showing that the median filter does not provide precise location of the jumps, due to the chosen time window of 300 msec. Lower trace shows the TVD filter used in the software for denoising the raw data and correct detection of the transition time point.

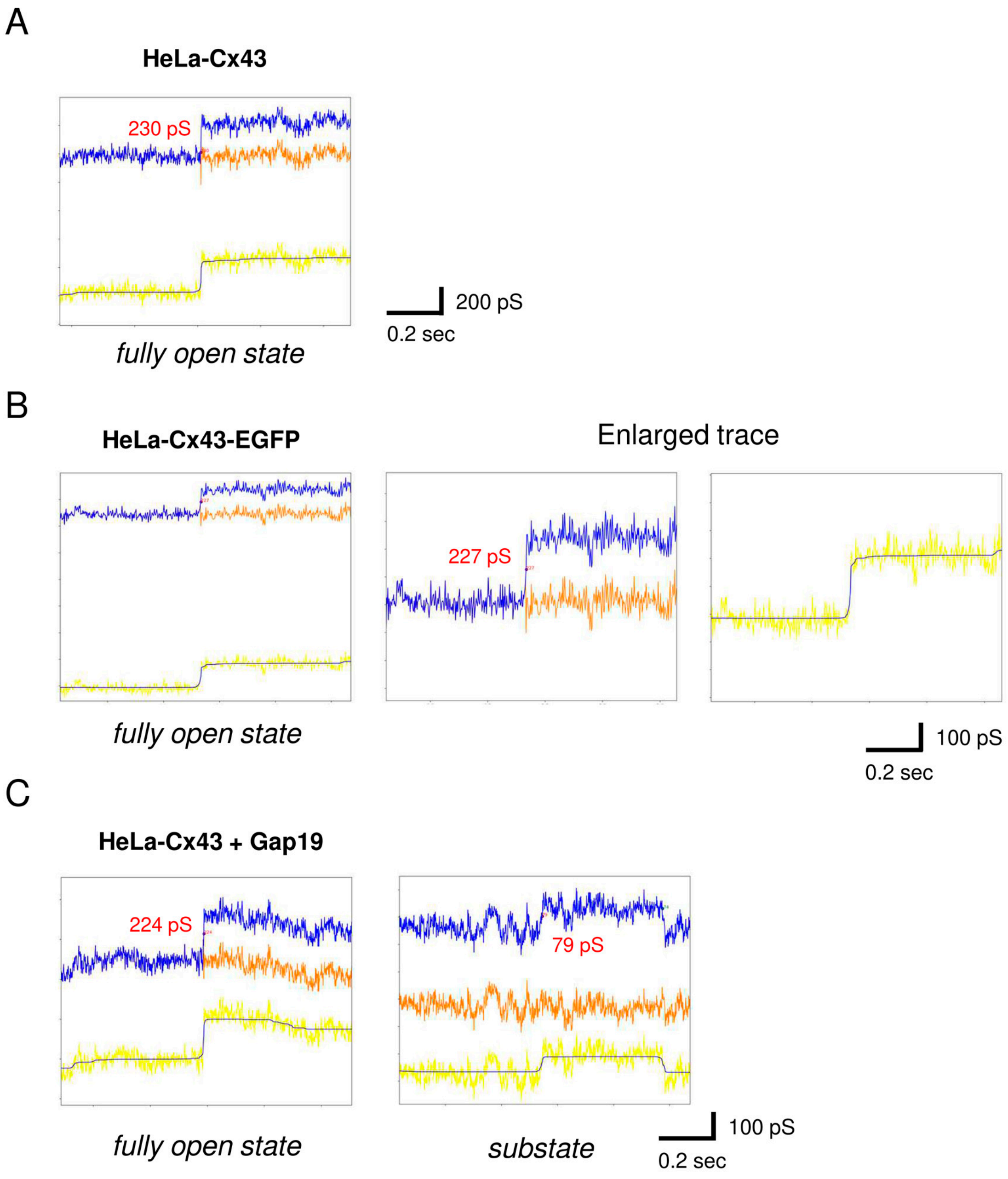

Supplementary Figure S3. TVD fitting of representative transitions obtained in three experimental conditions. A. HeLa-Cx43, **B.** HeLa-Cx43-EGFP and **C.** HeLa-Cx43 + Gap19 where the subconductance state is more prominent compared to the other conditions. The blue traces represent the raw data, the baseline is shown in orange and the yellow trace below is the baseline-corrected trace after the fitting. The blue line overlaying the yellow trace represents the signal after TVD filtering that was used for transition detection.

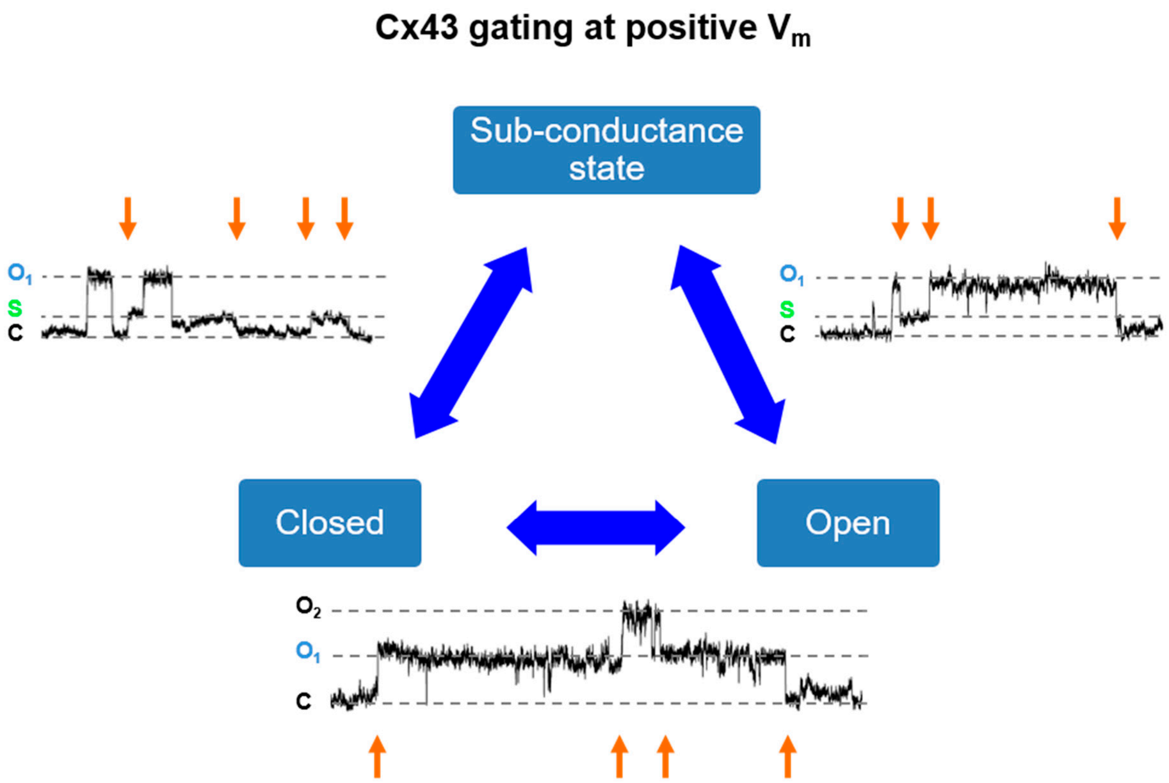

Supplementary Figure S4. Schematic representation of gating states observed in this study. Channel activity showed a closed state (C), a fully open state (O, main state) of ≈ 217 pS and a subconductance state (S) of ≈ 80 pS. Arrows highlight transitions between different states in exemplary traces shown. Transitions were observed between all three states.
